# Information Retrieval and Graph Analysis Approaches for Book Recommendation

**DOI:** 10.1155/2015/926418

**Published:** 2015-09-30

**Authors:** Chahinez Benkoussas, Patrice Bellot

**Affiliations:** ^1^Aix-Marseille Université, CNRS, LSIS UMR 7296, 13397 Marseille, France; ^2^Aix-Marseille Université, CNRS, CLEO OpenEdition UMS 3287, 13451 Marseille, France

## Abstract

A combination of multiple information retrieval approaches is proposed for the purpose of book recommendation. In this paper, book recommendation is based on complex user's query. We used different theoretical retrieval models: probabilistic as InL2 (Divergence from Randomness model) and language model and tested their interpolated combination. Graph analysis algorithms such as PageRank have been successful in Web environments. We consider the application of this algorithm in a new retrieval approach to related document network comprised of social links. We called Directed Graph of Documents (DGD) a network constructed with documents and social information provided from each one of them. Specifically, this work tackles the problem of book recommendation in the context of INEX (Initiative for the Evaluation of XML retrieval) Social Book Search track. A series of reranking experiments demonstrate that combining retrieval models yields significant improvements in terms of standard ranked retrieval metrics. These results extend the applicability of link analysis algorithms to different environments.

## 1. Introduction

In recent years, document retrieval and recommendation have become more and more popular in many Web 2.0 applications where user can request documents. There has been much work done both in the industry and academia on developing new approaches to improve the performance of retrieval and recommendation systems over the last decade. The interest in this area still remains high to help users to deal with information overload and provide recommendation or retrieval content (books, restaurants, movies, academic publications, etc.). Moreover, some of the vendors have incorporated recommendation capabilities into their commerce services, for example, Amazon in book recommendation.

Existing document retrieval approaches need to be improved to satisfy user's information needs. Most systems use classic information retrieval models, such as language models or probabilistic models. Language models have been applied with a high degree of success in information retrieval applications [1–3]. This was first introduced by Ponte and Croft in [4]. They proposed a method to score documents, called* query likelihood*. It consists of two steps: estimate a language model for each document and then rank documents according to the likelihood scores resulting from the estimated language model. Markov Random Field model was proposed by Metzler and Croft in [5]; it considers query term proximity in documents by estimating term dependencies in the context of language modeling approach. From the existing probabilistic models, InL2 a Divergence from Randomness-based model was proposed by Amati and Van Rijsbergen in [6]. It measures the global informativeness of the term in the document collection. It is based on the idea that “*the more the term occurrences diverge from random throughout the collection, the more informative the term is.*” The limit of such models is that the distance between query terms in documents is not considered.

In this paper, we present an approach that combines probabilistic and language models to improve the retrieval performances and show that the two models combined act much better in the context of book recommendation.

In recent years, an important innovation in information retrieval appeared which consists of algorithms developed to exploit the relationships between documents. One of the important algorithm is Google's PageRank [7]. It has been successful in Web environments, where the relationships are provided with the existing hyperlinks into documents. We present a new approach for document retrieval based on graph analysis and exploit the PageRank algorithm for ranking documents with respect to a user's query. In the absence of manually created hyperlinks, we use social information to create the Directed Graph of Documents (DGD) and argue that it can be treated in the same manner as hyperlink graphs. Experiments show that incorporating graph analysis algorithms in document retrieval improves the performances in term of the standard ranked retrieval metrics.

Our work focuses on search in the book recommendation domain, in the context of INEX Social Book Search track. The document collection contains Amazon/LibraryThing book descriptions and the queries, called topics, are extracted from the LibraryThing discussion forums.

In the rest of the paper, we presented a summary of related work in document retrieval and recommender systems. Then, we describe briefly the used retrieval models and show the combination method. In Section 5, we illustrate the graph modeling method followed by the different experiments and results.

## 2. Related Work

This work is first related to the area of document retrieval models, more specially language models and probabilistic models. The unigram language models are the most used for ad hoc information retrieval work; several researchers explored the use of language modeling that captures higher order dependencies between terms. Bouchard and Nie in [9] have showed significant improvements of retrieval effectiveness with a new statistical language model for the query based on three different ways: completing the query by terms in the user's domain of interest, reordering the retrieval results, or expanding the query using lexical relations extracted from the user's domain of interest.

Divergence from Randomness (DFR) is one of several probabilistic models that we have used in our work. Abolhassani and Fuhr have investigated several possibilities for applying Amati's DFR model [6] for content-only search in XML documents [10].

This work also relates to the category of graph based document retrieval. There has been increasing use of techniques based on graphs constructed by implicit relationships between documents. Kurland and Lee performed structural reranking based on centrality measures in graph of documents which has been generated using relationships between documents based on language models [11]. In [12], Lin demonstrates the possibility to exploit document networks defined by automatically generated content-similarity links for document retrieval in the absence of explicit hyperlinks. He integrates the PageRank scores with standard retrieval score and shows a significant improvement in ranked retrieval performance. His work was focused on search in the biomedical domain, in the context of PubMed search engine.

## 3. INEX Social Book Search Track and Test Collection

SBS task (http://social-book-search.humanities.uva.nl/) aims to evaluate the value of professional and user's metadata for book search on the Web. The main goal is to exploit search techniques to deal with complex information needs and complex information sources that include user profiles, personal catalogs, and book descriptions.

The SBS task builds on a collection of 2.8 million book description crawled by the University of Duisburg-Essen from Amazon (http://www.amazon.com/) [13] and enriched with content from LibraryThing (http://www.librarything.com/). Each book is identified by an ISBN and is an XML file. It contains content information like title information, Dewey Decimal Classification (DDC) code (for 61% of the books), category, Amazon product description, and so forth. Amazon records contain also social information generated by users like tags, reviews, and ratings. For each book, Amazon suggests a set of “Similar Products” which represents a result of computed similarity based on content information and user behavior (purchases, likes, reviews, etc.) [14].

SBS task provides a set of queries called topics each year where users describe what they are looking for (books for a particular genre, books of particular authors, similar books to those that have been already read, etc.). These requests for recommendations are natural expressions of information needs for a large collection of online book records. The topics are crawled from LibraryThing discussion forums.

**Figure 1:**
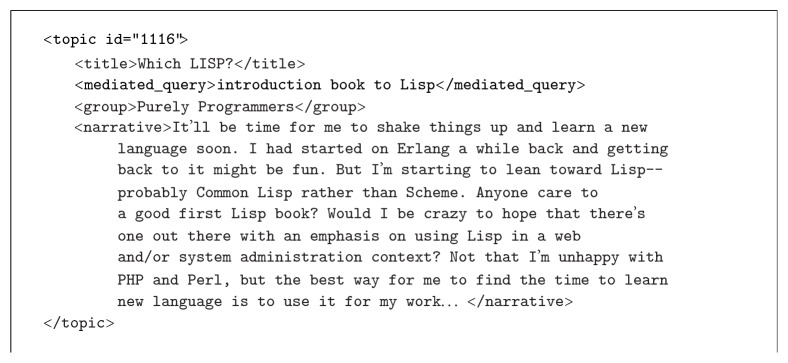
An example of topic from SBS topics set composed with multiple fields to describe user's need.

The topic set consists of 680 topics and 208 topics in 2014 and 2015, respectively. Each topic has a narrative description of the information needs. The topic set of 2015 is a subset of that of 2014. Each topic consists of a set of fields. In this contribution we use* title*,* mediated query* (query description), and* narrative* fields. An example of topic is illustrated in Figure 1.

## 4. Retrieval Models

This section presents brief description and combination method of retrieval models used for book recommendation.

### 4.1. InL2 of Divergence from Randomness

We used InL2, Inverse Document Frequency model with Laplace after-effect and Normalization 2. This model has been used with success in different works [15–18]. InL2 is a DFR-based model (Divergence from Randomness) based on the Geometric distribution and Laplace law of succession. The DFR models are based on this idea: “The more the divergence of the within-document term-frequency from its frequency within the collection, the more the information carried by the word *t* in the document *d*” [19]. For this model, the relevance score of a document *D* for a query *Q* is given by

(1) $$\begin{array}{c} \text{score}\left(\;Q,D\;\right)=\underset{t\in Q}{\sum }\text{qtw}\cdot \frac{1}{\text{tfn}+1}\left(\;\text{tfn}\cdot \log\frac{N+1}{n_{t}+0.5}\;\right), \end{array}$$

where qtw is the query term weight given by qtf/qtf_max⁡_; qtf is the query term frequency; qtf_max⁡_ is the maximum query term frequency among the query terms. *N* is the number of documents in the whole collection and *n*_*t*_ is the number of documents containing *t*. tfn corresponds to the weighted sum of normalized term frequencies tf_*f*_ for each field *f*, known as* Normalization 2* and given by

(2) $$\begin{array}{c} \text{tfn}=\text{tf}\cdot \log\left(\;1+c\cdot \frac{\text{avg\_}l}{l}\;\right)\;\left(c>0\right), \end{array}$$

where tf is the frequency of term *t* in the document *D*; *l* is the length of the document in tokens and avg_*l* is the average length of all documents; *c* is a hyperparameter that controls the normalization applied to the term frequency with respect to the document length.

### 4.2. Sequential Dependence Model of Markov Random Field

Language models are largely used in document retrieval search for book recommendation [16, 20]. Metzler and Croft's Markov Random Field (MRF) model [21, 22] integrates multiword phrases in the query. Specifically, we used the Sequential Dependence Model (SDM), which is a special case of MRF. In this model cooccurrence of query terms is taken into consideration. SDM builds upon this idea by considering combinations of query terms with proximity constraints which are single term features (standard unigram language model features, *f*_*T*_), exact phrase features (words appearing in sequence, *f*_*O*_), and unordered window features (require words to be close together, but not necessarily in an exact sequence order, *f*_*U*_).

Finally, documents are ranked according to the following scoring function:

(3) SDMQ,D=λT∑q∈QfTq,D+λO∑i=1Q−1fOqi,qi+1,D+λU∑i=1Q−1fUqi,qi+1,D,

where feature weights are set based on the author's recommendation (*λ*_*T*_ = 0.85, *λ*_*O*_ = 0.1, *λ*_*U*_ = 0.05) in [20]. *f*_*T*_, *f*_*O*_, and *f*_*U*_ are the log maximum likelihood estimates of query terms in document *D* as shown in Table 1, computed over the target collection using a Dirichlet smoothing. We applied this model to the queries using Indri (http://www.lemurproject.org/indri/) Query Language (http://www.lemurproject.org/lemur/IndriQueryLanguage.php).

### 4.3. Combining Search Systems

The use of different retrieval systems retrieves different sets of documents. Combining the output of many search systems, in contrast to using just a single retrieval technique, can improve the retrieval effectiveness as shown by Belkin et al. in [23] where the authors have combined the results of probabilistic and vector space models. In our work, we combined the results of InL2 model and SDM model. The retrieval models use different weighting schemes; therefore we should normalize the scores. We used the maximum and minimum scores according to Lee's formula [24] as follows:

(4) $$\begin{array}{c} normalizedScore=\frac{old\text{Score}-min\text{Score}}{max\text{Score}-min\text{Score}}. \end{array}$$

It has been shown in [16] that InL2 and SDM models have different levels of retrieval effectiveness; thus it is necessary to weight individual model scores depending on their overall performance. We used an interpolation parameter (*α*) that we have varied to get the best interpolation that provides better retrieval effectiveness.

## 5. Graph Modeling

We have studied the INEX SBS collection to link documents. In [25], the authors have used PubMed collection and exploited networks defined by automatically generated content-similarity links for document retrieval. In our case, we exploited a special type of similarity based on several factors. This similarity is provided by Amazon and corresponds to “Similar Products” given generally for each visited book. Amazon suggests books in “Similar Products” according to there similarity to the consulted book. The degree of similarity is relative to user's social information like clicks or purchases and content based information like book attributes (book description, book title, etc.). The exact formula that combines social and content based information to compute similarity is not delivered by Amazon.

To perform data modeling into DGD, we extracted the “Similar Products” links between documents. The constructed DGD will be used to enrich returned results by the retrieval models; that is, we use the graph structure in the same spirit of pseudorelevance-feedback algorithms. Our method can potentially serve to greatly enhance the retrieval performances.

In this section we use some fixed notations. The collection of documents is denoted by *C*. In *C*, each document *d* has a unique ID. The set of queries called topics is denoted by *T*, the set *D*_init_ ⊂ *C* refers to the documents returned by the initial retrieval model. StartingNode indicates document from *D*_init_ which is used as input to graph processing algorithms in the DGD. The set of documents present in the graph is denoted by *S*. *D*_*t*_*i*__ indicates the documents retrieved for topic *t*_*i*_ ∈ *T*.

Each node in the DGD represents document (Amazon description of book) and has set of properties:

(i) ID: book's ISBN;
(ii) content: book description that includes many other properties (title, product description, author(s), users' tags, content of reviews, etc.);
(iii) MeanRating: average of ratings attributed to the book;
(iv) PR: value of book PageRank.

**Figure 2:**
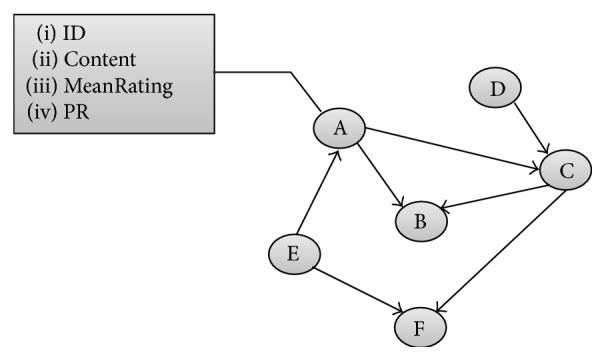
Example of Directed Graph of Documents.

Nodes are connected with directed links; given nodes {*A*, *B*} ∈ *S*, if *A* points to *B*, *B* is suggested as Similar Product to *A*. In Figure 2, we show an example of DGD, network of documents. The DGD network contains 1.645.355 nodes (89.86% of nodes are in the collection and the rest do not belong to it) and 6.582.258 relationships.

### 5.1. Our Approach

The DGD network contains useful information about documents that can be exploited for document retrieval. Our approach is based first on results of traditional retrieval approach and then on the DGD network to find other documents. The idea is to suppose that the suggestions given by Amazon can be relevant to the user queries.

We introduce the algorithm called “retrieving based on DGD feedback.” Algorithm 1 takes the following as inputs: *D*_init_ returned list of documents for each topic by the retrieval techniques described in Section 3, DGD network, and parameter *β* which is the number of the top selected StartingNode from *D*_init_ denoted by *D*_StartingNodes_. We fixed *β* to 100 (10% of the returned list for each topic). The algorithm returns list of recommendations for each topic denoted by “*D*_final_”. It processes topic by topic and extracts the list of all neighbors for each StartingNode. It performs mutual Shortest Paths computation between all selected StartingNode in DGD. The two lists (neighbors and nodes in computed Shortest Paths) are concatenated; after that all duplicated nodes are deleted. The set of documents in returned list is denoted by “*D*_graph_”. A second concatenation is performed between initial list of documents and *D*_graph_ (all duplications are deleted) in new final list of retrieved documents; “*D*_final_” reranked using different reranking schemes.

**Figure 3:**
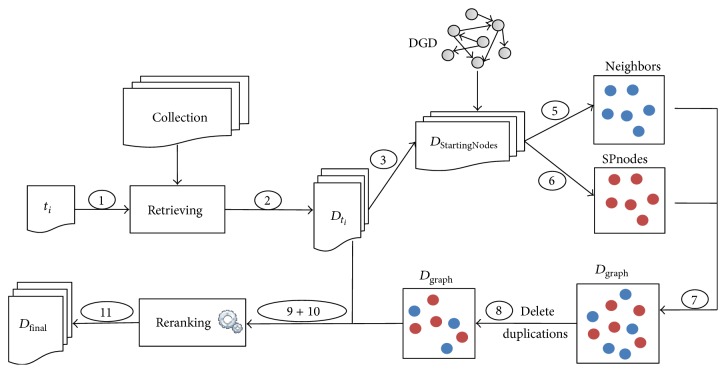
Architecture of book retrieval approach based on DGD feedback.

Figure 3 shows the architecture of the document retrieval approach based on DGD feedback. The numbers on arrows represent instructions in Algorithm 1.

## 6. Experiments and Results

In this section, we describe the experimental setup we used for our experiments. Furthermore, we present the different reranking schemes used in previously defined approaches.

### 6.1. Experiments Setup

For our experiments, we used different tools that implement retrieval models and handle the graph processing. First, we used Terrier (Terabyte Retriever) (http://terrier.org/) Information Retrieval framework developed at the University of Glasgow [26–28]. Terrier is a modular platform for rapid development of large-scale IR applications. It provides indexing and retrieval functionalities. It is based on DFR framework and we used it to deploy InL2 model described in Section 4.1. Further information about Terrier can be found at http://ir.dcs.gla.ac.uk/terrier.

A preprocessing step was performed to convert INEX SBS corpus into Trec Collection Format (http://lab.hypotheses.org/1129), by considering that the content of all tags in each XML file is important for indexing; therefore the whole XML file was transformed on one document identified by its ISBN. Thus, we just need two tags instead of all tags in XML, the ISBN and the whole content (named text).

Secondly,* Indri *(http://www.lemurproject.org/indri/)*, Lemur Toolkit for Language Modeling and Information Retrieval* was used to carry out a language model (SDM) described in Section 4.2. Indri is a framework that provides state-of-the-art text search methods and a rich structured query language for big collections (up to 50 million documents). It is a part of the Lemur project and developed by researchers from UMass and Carnegie Mellon University. We used Porter stemmer and performed Bayesian smoothing with Dirichlet priors (Dirichlet prior *μ* = 1500).

In Section 5.1, we have described our approach based on DGD which includes graph processing. We used NetworkX (https://networkx.github.io/) tool of Python to perform shortest path computing, neighborhood extraction, and PageRank calculation.

To evaluate the results of retrieval systems, several measurements have been used for SBS task: Discounted Cumulative Gain (nDCG), the most popular measure in IR [29], Mean Average Precision (MAP) which calculates the mean of average precisions over a set of queries, and other measures: Recip Rank and Precision at the rank 10 (P@10).

### 6.2. Reranking Schemes

**Figure 4:**
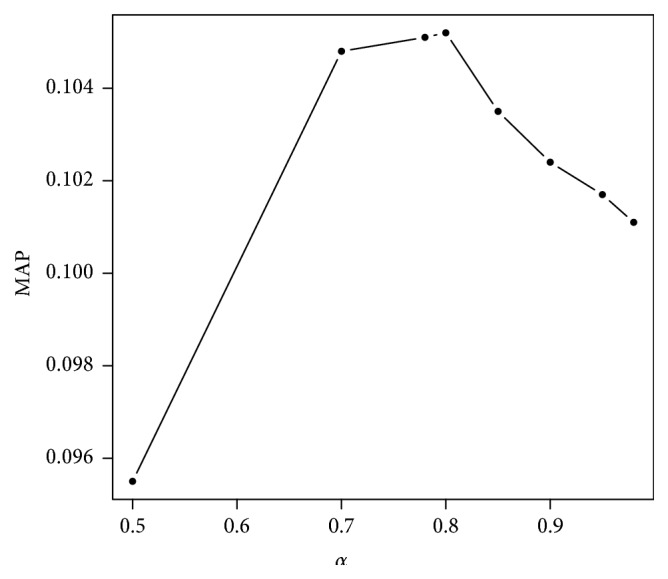
MAP distribution for INEX SBS 2014 when varying the interpolation parameter *α*.

In this paper, we have proposed two approaches. The first (see Section 4.3) consists of merging the results of two different information retrieval models which are the language model (SDM) and DFR model (InL2). For topic *t*_*i*_, each of the models gives 1000 documents and each retrieved document has an associated score. The linear combination method uses the following formula to calculate final score for each retrieved document *d* by SDM and InL2 models:

(5) finalscored,ti=α·scoreInL2d,ti+1−α·scoreSDMd,ti,

where score_InL2_(*d*, *t*_*i*_) and score_SDM_(*d*, *t*_*i*_) are normalized scores. *α* is the interpolation parameter set up at 0.8 after several tests on topics 2014, according to the MAP measure shown in Figure 4.

The second approach (described in Section 5.1) uses the DGD constructed from the “Similar Products” information. The document set returned by the retrieval model are fused to the documents in neighbors set and Shortest Path results. We tested different reranking methods that combine the retrieval model scores and other scores based on social information. For each document in the resulting list, we calculated the following scores:

- *PageRank*, computed using NetworkX tool: it is a well-known algorithm that exploits link structure to score the importance of nodes in a graph. Usually, it has been used for hyperlink graphs such as the Web [30].
- *MeanRatings*, information generated by users: it represents the mean of all ratings attributed by users for a book.

The computed scores were normalized using this formula: normalizedScore = oldScore/maxScore. After that, to combine the results of retrieval system and each of normalized scores, an intuitive solution is to weight the retrieval model scores with the previously described scores (normalized PageRank and MeanRatings). However, this would favor documents with high PageRank and MeanRatings scores even though their content is much less related to the topics.

### 6.3. Results

In this section, we discuss the results we achieved by using the InL2 retrieval model, its combination to the SDM model, and retrieval system proposed in our approach that uses the graph structure DGD.

We used two topic sets provided by INEX SBS task in 2014 (680 topics) and 2015 (208 topics). The systems retrieve 1000 documents per topic. The experimental results, which describe the performance of the different retrieval systems on INEX document collection, are shown in Table 2.

As illustrated in Table 2, the system that combines probabilistic model InL2 and the language model SDM (InL2_SDM) achieves a significant improvement comparing to InL2 model (baseline). The two systems do not provide similar level of retrieval effectiveness (as proved in [16]). The results of run InL2_DDG_PR using the 2015 topic set confirm that incorporating PageRank scores using our approach based on DGD network improves ranked retrieval performance but decreases the baseline performances when using the 2014 topic set. The run that uses the MeanRatings property (InL2_DGD_MnRtg) to rerank retrieved documents is the lowest performer in terms of all measurements for 2014 topic set. That means that ratings given by users do not help to improve the reranking performances for 2014 topic set.

Notice that 2015 topic set contains a subset of 2014 topic set. We can clearly observe the difference between results of systems using the two topic sets. We think that the main reason is the evaluation processes that are not the same. Where analysing the qrels of common topics between 2014 and 2015 sets, we found that relevancy values are not the same in most cases. More details on the evaluation processes used in 2014 and 2015 can be found in [31] and http://social-book-search.humanities.uva.nl/#/suggestion.

Nevertheless, the depicted results confirm that we are starting with competitive baseline, the improvements contributed by combining the retrieval systems' outputs and social link analysis are indeed meaningful.

## 7. Conclusion and Future Work

This paper proposed and evaluated two approaches of document retrieval in the context of book recommendation. We used the test collection of INEX Social Book Search track and the proposed topics in 2014 and 2015. We presented the first approach that combines the outputs of probabilistic model (InL2) and language model (SDM) using a linear interpolation after normalizing scores of each retrieval system. We have shown a significant improvement of baseline results using this combination.

This paper also proposed a novel approach based on Directed Graph of Documents (DGD). It exploits social link structure to enrich the returned document list by traditional retrieval model (InL2, in our case). We performed a reranking method using PageRank and ratings of each retrieved document.

For future work, we would like to test the proposed approaches in this paper on another test collection which consists of OpenEdition Portal. It is dedicated to electronic resources in the humanities and social sciences. We would like to explore citation links between scientific documents extracted using Kim et al. [32]. Using the traversal algorithms we will develop a new way to retrieve documents. Another interesting extension of this work would be using the learning to rank techniques to automatically adjust the settings of reranking parameters.

## Figures and Tables

**Algorithm 1:**
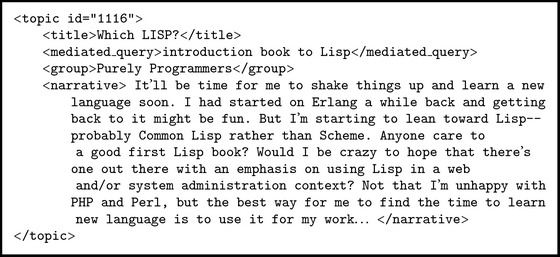
Retrieving based on DGD feedback.

**Table 1:** Language modeling-based unigram and term weighting functions. Here, *tf*_*e*,*D*_ is the number of times term *e* matches in document *D*, *cf*_*e*,*D*_ is the number of times term *e* matches in the entire collection, |*D*| is the length of document *D*, and |*C*| is the size of the collection. Finaly, μ is a weighting function hyperparameter that is set to 2500.

Weighting	Description
$f_{T}\left(q_{i},D\right)=\log\left[\frac{tf_{q_{i},D}+\mu cf_{q_{i}}/\left|C\right|}{\left|D\right|+\mu }\right]$	Weight of unigram *q*_*i*_ in document *D*.

$f_{O}\left(q_{i},q_{i+1},D\right)=\log\left[\frac{tf_{\#1\left(q_{i},q_{i+1}\right),D}+\mu cf_{\#1\left(q_{i},q_{i+1}\right)}/\left|C\right|}{\left|D\right|+\mu }\right]$	Weight of exact phrase “*q*_*i*_ *q*_*i*+1_” in document *D*.

$f_{O}\left(q_{i},q_{i+1},D\right)=\log\left[\frac{tf_{\#uw8\left(q_{i},q_{i+1}\right),D}+\mu cf_{\#uw8\left(q_{i},q_{i+1}\right)}/\left|C\right|}{\left|D\right|+\mu }\right]$	Weight of unordered window “*q*_*i*_ *q*_*i*+1_” (span = 8) in document *D*.

**Table 2:** Experimental results. The runs are ranked according to nDCG@10. (*∗*) denotes significance according to Wilcoxon test [8]. In all cases, all of our tests produced two-sided *p* value, α = 0.05.

2014 topic set				
Run	nDCG@10	Recip Rank	MAP	P@10
**InL2**	**0.128**	**0.236**	**0.101**	**0.067**
InL2_SDM	0.136 (+6%^*∗*^)	0.249 (+5%^*∗*^)	0.1052 (+4%^*∗*^)	0.070 (+4%^*∗*^)
InL2_DGD_PR	0.122 (−4%^*∗*^)	0.239 (+1%^*∗*^)	0.090 (−9%^*∗*^)	0.0695 (+2%^*∗*^)
InL2_DGD_MnRtg	0.105 (−17%^*∗*^)	0.192 (−18%^*∗*^)	0.081 (−18%^*∗*^)	0.057 (−15%^*∗*^)

2015 topic set				
Run	nDCG@10	Recip Rank	MAP	P@10

**InL2**	**0.063**	**0.147**	**0.046**	**0.044**
InL2_SDM	0.069 (+9%^*∗*^)	0.166 (+12%^*∗*^)	0.051 (+10%)	0.050 (+13%^*∗*^)
InL2_DGD_PR	0.068 (+7%^*∗*^)	0.157 (+6%^*∗*^)	0.048 (+4%^*∗*^)	0.052 (+18%^*∗*^)
InL2_DGD_MnRtg	0.066 (+4%)	0.148 (+0.6%)	0.042 (−8%)	0.052 (+18%^*∗*^)
